# PNPLA6‐Related Disorder with Levodopa‐Responsive Parkinsonism

**DOI:** 10.1002/mdc3.13632

**Published:** 2022-12-14

**Authors:** Serdar Kazanci, Jennifer Witt, Kimmy Su, Oswaldo Lorenzo‐Betancor, Dora Yearout, Cyrus P. Zabetian, Marie Y. Davis

**Affiliations:** ^1^ Department of Neurology University of Washington Medical Center Seattle WA USA; ^2^ Booth Gardner Parkinson's Care Center Evergreen Hospital Medical Center Kirkland WA USA; ^3^ VA Puget Sound Healthcare System Seattle WA USA

**Keywords:** PNPLA‐6, hereditary spastic paraplegia type 39, HSP39, parkinsonism

The *PNPLA6* gene encodes neuropathy target esterase (NTE) which is expressed in the eye, brain, and pituitary gland. NTE was first characterized as a target of organophosphorous esters, causing delayed‐onset axonal degeneration.^1^, ^2^ *PNPLA6*‐related disorders are autosomal recessive, with heterogeneous manifestations, including cerebellar ataxia, upper motor neuron dysfunction, chorioretinal dystrophy, hypogonadotropic hypogonadism with or without anterior hypopituitarism, peripheral neuropathy, hair anomalies, short stature, and cognitive impairment. Several associated syndromes are recorded in the Online Mendelian Inheritance in Man® including hereditary spastic paraplegia type (HSP) 39 (OMIM 612020). Two individuals with bi‐allelic variants in *PNPLA6* have been reported with Parkinsonian features.^3^ Our case further supports levodopa‐responsive parkinsonism due to *PNPLA6* variants.

A 45‐year‐old male of European and Ashkenazi Jewish ancestry presented with a 6‐year history of progressive intermittent tremor in the left shoulder and head with occasional spread to the right upper limb. Additional complaints were micrographia and slurred speech.

Past medical history included motor delay since infancy, with stiff legs and difficulty running throughout childhood. Dorsal rhizotomy at the age of 16 significantly improved lower extremity spasticity but caused hypotonia and loss of independent ambulation. There was no evidence of intellectual disability or cognitive decline.

Family history was significant for two full siblings, a healthy 3 years younger sister, and an 11 years younger brother. The brother is normal in height (182.9 cm) with gait ataxia requiring use of a wheelchair, obesity, intellectual disability, cryptorchidism, and low testosterone. The parents are healthy. There is no known consanguinity, and no additional report of neurologic symptoms in a three‐generation pedigree.

Exam was abnormal for short stature (157.5 cm), vertical and horizontal gaze‐evoked nystagmus, and mild dysarthria. Tone and muscle strength were severely decreased in the lower extremities. Deep tendon reflexes were brisk in the upper extremities and absent in the lower extremities. A “no‐no” head tremor and left upper limb tremor involving the shoulder and hand were intermittently present at rest and subsided with action. The tremor was regular and approximately ~6 Hz, maximum amplitude ~1 cm. Slight cogwheel rigidity was detected in the left arm. Moderate bradykinesia was observed with finger tapping, hand opening and closing, and pronation‐supination movements in the left arm, and slight bradykinesia in the right arm. Mild dysmetria was noted in the left hand. Other features of *PNPLA6* related syndromes such as chorioretinal dystrophy, peripheral neuropathy, and hypogonadotropic hypogonadism were absent. Brain MRI showed midline cerebellar atrophy on sagittal T1 (Fig. 1A).

**FIG. 1:**
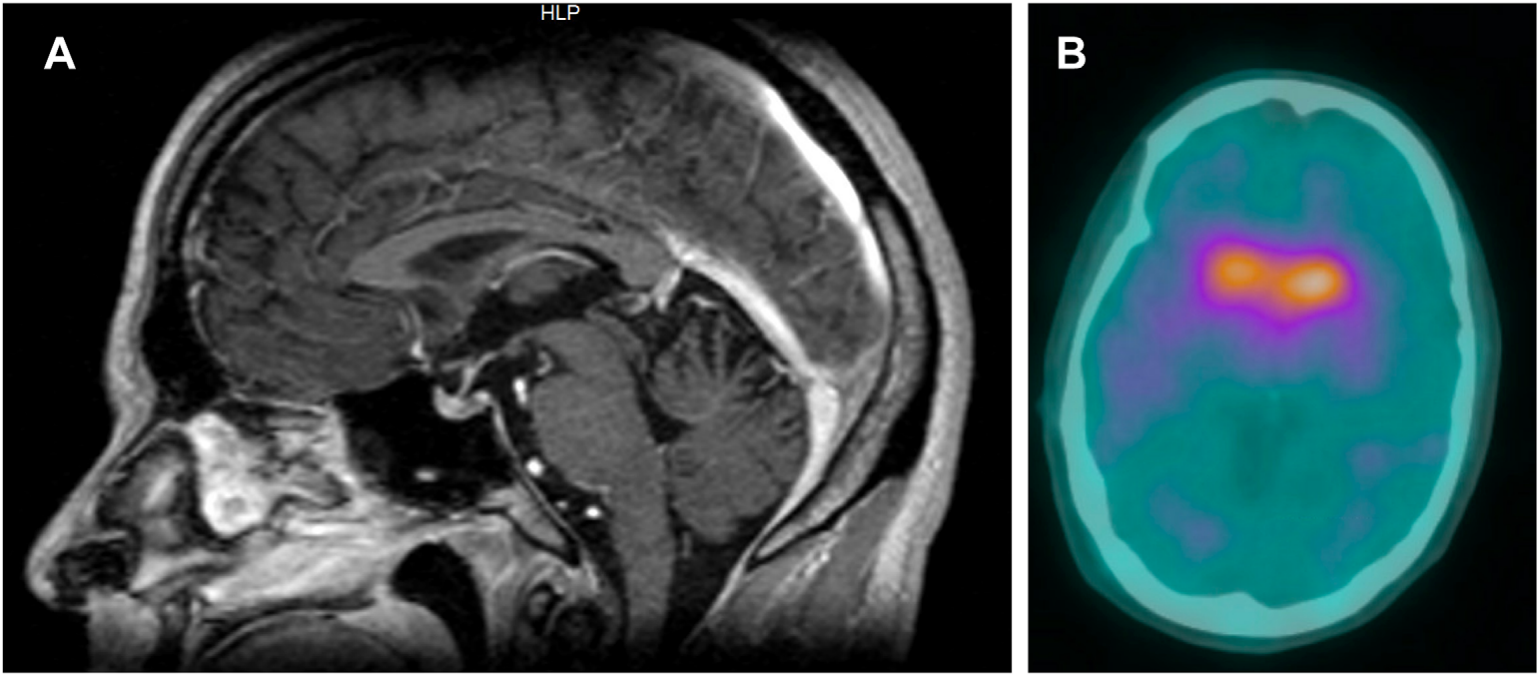
MRI brain and Ioflupane I‐123 SPECT imaging. (A) Brain MRI sagittal T1 demonstrated midline cerebellar atrophy. (B) Ioflupane I‐123 SPECT imaging shows decreased radiotracer uptake within the posterior putamen bilaterally.

Examination of the brother revealed horizontal and vertical gaze evoked nystagmus, mild dysarthria, mild bradykinesia with bilateral finger tapping, and increased tone in the lower extremities. No resting, postural, or kinetic tremor was noted.

A next generation clinical sequencing panel of 24 genes causing autosomal recessive HSPs (Invitae) was performed on the proband. This revealed two variants within the *PNPLA6* locus: pathogenic variant NM_001166114.2:c.3058_3061dup (p.Arg1021fs) (ClinVar VCV000006607.29, accessed 10/19/2022),^4^ and variant of uncertain significance NM_001166114.2:c.4003C > T (p.Pro1335Ser) (ClinVar VCV000409994.8, accessed October 19, 2022, see Supplementary Table S1). Subsequent segregation analysis revealed that the patient's mother was heterozygous for c.3058_3061dup and father was heterozygous for c.4003C > T. The patient's brother also carried both *PNPLA6* variants, while his unaffected sister carried only c.3058_3061dup. Since the phenotype included parkinsonism, a panel including 26 PD‐related genes (Supplementary Table S1) was sequenced, and no pathogenic single nucleotide or copy number variants were identified in any of these genes

Following a trial of 100 mg levodopa twice per day, the patient's resting tremor was infrequent, and bradykinesia improved to slight or mild in the upper extremities (Supplementary on–off video 1). Ioflupane I‐123 SPECT imaging showed symmetric decreased radiotracer uptake within the posterior putamen bilaterally, consistent with nigrostriatal degeneration (Fig. 1B).

**Video 1:** Video sequence of proband examined off levodopa therapy followed by examination 30 minutes after taking two tabs of immediate release carbidopa/levodopa 25 mg/100 mg.

*PNPLA6* variants cause a broad range of phenotypes, including HSP, ataxia, retinal, neuroendocrine, and cognitive manifestations. Our case adds to a prior report of levodopa‐responsive parkinsonism, and provides further evidence supporting the pathogenicity of *PNPLA6* variant c.4003C > T (p.Pro1335Ser). Sen et al. (2020) reported two siblings with biallelic *PNPLA6* variants who developed levodopa responsive parkinsonism and HSP in their 4th or 5th decade of life without additional non‐motor manifestations that can occur with *PNPLA6* variants. Our patient manifested HSP symptoms as well as levodopa‐responsive parkinsonism, short stature, and ataxia.

*PNPLA6* is highly conserved from yeasts to mammals, encoding a phospholipase that is localized to the cytoplasmic surface of the endoplasmic reticulum. Model organisms with loss of function of NTE indicate that its phospholipase activity is important for intracellular phospholipid homeostasis, membrane phospholipid trafficking, axonal maintenance, mitochondrial dynamics, and mitigating oxidative stress.^4^, ^5^, ^6^ Defects in many of these mechanisms have already been implicated in the pathogenesis of parkinsonism,^7^, ^8^, ^9^, ^10^ making the manifestation of early onset parkinsonism in the spectrum of *PNPLA6*‐related phenotypes not unexpected.

This case further supports the manifestation of treatment‐responsive parkinsonism with *PNPLA6* variants. Further model organism and clinical studies are needed to elucidate the multiplex of *PNPLA6*‐associated syndromes.

## Author Roles

(1) Research project: A. Conception, B. Organization, C. Execution (2) Statistical Analysis: A. Design, B. Execution, C. Review and Critique (3) Manuscript Preparation: A. Writing of the first draft, B. Review and Critique.

JW: 1A, 1B, 1C, 3B.

KS: 1B, 1C, 3A.

OLB: 1C, 3B.

DY: 1C, 3B.

CPZ: 1C, 3B.

MYD: 1A, 1B, 1C, 3B.

## Disclosures

**Ethical Compliance Statement:** IRB approval was obtained from the University of Washington (IRB ID STUDY00011942) and VA Puget Sound (IRBNet ID # 1587534). Declaration of patient consent: Written consent documented on electronic medical record. We confirm that we have read the Journal's position on issues involved in ethical publication and affirm that this work is consistent with those guidelines. The contents do not represent the views of the U.S. Department of Veterans Affairs or the United States Government.

**Funding Sources and Conflicts of Interest:** This work was supported by grants from the Department of Veterans Affairs (I01 CX001702 to CPZ and IK2 BX003244 to MYD). The authors declare that there are no conflicts of interest relevant to this work.

**Financial Disclosures for the previous 12 months:** SK, JW, KS, OLB, DY, CPZ and MYD declare that there are no additional disclosures to report.

## Supporting information


**Appendix S1.** Supporting Information.

**Table S1.** Rare variant in PD‐related genes and *PNPLA6* variants identified in the familial proband.

## References

[1] Pope CN , Tanaka D J , Padilla S . The role of neurotoxic esterase (NTE) in the prevention and potentiation of organophosphorus‐induced delayed neurotoxicity (OPIDN). Chem Biol Interact 1993 Jun;87(1–3):395–406. 8343996 10.1016/0009-2797(93)90067-9

[2] Richardson RJ , Fink JK , Glynn P , Hufnagel RB , Makhaeva GF , Wijeyesakere SJ . Neuropathy target esterase (NTE/PNPLA6) and organophosphorus compound‐induced delayed neurotoxicity (OPIDN). Adv Neurotoxicol 2020;4:1–78. 32518884 10.1016/bs.ant.2020.01.001 PMC7271139

[3] Sen K , Finau M , Ghosh P . Bi‐allelic variants in PNPLA6 possibly associated with parkinsonian features in addition to spastic paraplegia phenotype. J Neurol 2020;267(9):2749–2753. 32623594 10.1007/s00415-020-10028-w

[4] Hufnagel RB , Arno G , Hein ND , et al. Neuropathy target esterase impairments cause Oliver‐McFarlane and Laurence‐moon syndromes. J Med Genet 2015;52(2):85–94. 25480986 10.1136/jmedgenet-2014-102856 PMC8108008

[5] Akassoglou K , Malester B , Xu J , Tessarollo L , Rosenbluth J , Chao MV . Brain‐specific deletion of neuropathy target esterase/swisscheese results in neurodegeneration. Proc Natl Acad Sci U S A 2004;101(14):5075–5080. 15051870 10.1073/pnas.0401030101 PMC387376

[6] Melentev PA , Ryabova EV , Surina NV , et al. Loss of swiss cheese in neurons contributes to neurodegeneration with mitochondria abnormalities, reactive oxygen species acceleration and accumulation of lipid droplets in drosophila brain. Int J Mol Sci 2021;22(15). 10.3390/ijms22158275 PMC8347196 34361042

[7] Trist BG , Hare DJ , Double KL . Oxidative stress in the aging substantia nigra and the etiology of Parkinson's disease. Aging Cell 2019;18(6):e13031. 31432604 10.1111/acel.13031 PMC6826160

[8] Mercado G , Castillo V , Soto P , Sidhu A . ER stress and Parkinson's disease: Pathological inputs that converge into the secretory pathway. Brain Res 2016;1648:626–632. 27103567 10.1016/j.brainres.2016.04.042

[9] Lin G , Wang L , Marcogliese PC , Bellen HJ . Sphingolipids in the pathogenesis of Parkinson's disease and parkinsonism. Trends Endocrinol Metab 2019;30(2):106–117. 30528460 10.1016/j.tem.2018.11.003

[10] Povysil G , Tzika A , Vogt J , et al. Panelcn.MOPS: Copy‐number detection in targeted NGS panel data for clinical diagnostics. Hum Mutat 2017;38(7):889–897. 28449315 10.1002/humu.23237 PMC5518446

