# Supplementary material for: PNPLA6‐Related Disorder with Levodopa‐Responsive Parkinsonism
Source: Mov Disord Clin Pract. 2022 Dec 14;10(2):338–40. doi: 10.1002/mdc3.13632 (PMC9941917; doi:10.1002/mdc3.13632)
Supplement: Supplementary file 1 — Appendix S1. Supporting Information. [file MDC3-10-338-s002.docx]

**Supplementary Methods**. Genomic DNA was extracted from peripheral blood using standard methods. The proband was screened using a targeted next generation sequencing panel which included 26 genes related to disorders in which parkinsonism is a central feature: *ATP13A2*, *ATP1A3*, *DNAJC13*, *DNAJC6*, *EIF4G1*, *FBXO7*, *GBA*, *GCH1*, *GIGYF2*, *HTRA2*, *LRRK2*, *PARK7*, *PINK1*, *PLA2G6*, *PRKN*, *RAB39B*, *SLC6A3*, *SNCA*, *SNCB*, *SYNJ1*, *TAF1*, *TARDBP*, *TH*, *TMEM230*, *VPS13C*, and *VPS35*. All these genes except *TAF1* and *RAB39B* (chromosome X genes), were screened for copy number variants (CNV) using a bioinformatic approach with the cnpanel.MOPS R package^10.^
