# Supplementary material for: PNPLA6‐Related Disorder with Levodopa‐Responsive Parkinsonism
Source: Mov Disord Clin Pract. 2022 Dec 14;10(2):338–40. doi: 10.1002/mdc3.13632 (PMC9941917; doi:10.1002/mdc3.13632)
Supplement: Supplementary file 2 — Table S1. Rare variant in PD‐related genes and PNPLA6 variants identified in the familial proband. [file MDC3-10-338-s001.docx]

**Supplementary Table 1.** Rare variant in PD-related genes and *PNPLA6* variants identified in the familial proband. cDNA = complementary DNA; CADD = Combined Annotation Dependent Depletion; ACMG = American College of Medical Genetics; D = deleterious; NA – not available. The threshold for rare variants was set below a minor allele frequency (MAF) of 0.01 in the Non-Finnish European population of gnomAD (Genome Aggregation Database) v.2.1.1. *Reported GnomAD MAF represents Non-Finnish European population.

| Gene | cDNA | Protein | Isoform used for annotation | Genotype | GnomAD MAF* | CADD score | Polyphen2 prediction | ClinVar | ACMG classification |
| --- | --- | --- | --- | --- | --- | --- | --- | --- | --- |
| *VPS13C* | c.2270A>G | p.Q757R | NM_020821 | 0/1 | 0.0007 | 25.2 | Probably damaging | Likely benign | Benign |
| *PNPLA6* | c.3058_3061dup | p.Arg1021fs | NM_001166114.2 | 0/1 | 0 | NA | NA | Pathogenic | Likely pathogenic |
| *PNPLA6* | c.4003C>T | p.Pro1335Ser | NM_001166114.2 | 0/1 | 0.000017 | 19.1 | Benign | Pathogenic | Likely benign |
